# Exploring the preparedness of hospital pharmacists practising in Southwest London for implementing pharmacogenomics testing

**DOI:** 10.1080/20523211.2024.2432460

**Published:** 2024-12-04

**Authors:** Thuy Mason, Éirne Ní Dheasmhúnaigh, Heba Ghazal

**Affiliations:** School of Life Sciences, Pharmacy and Chemistry, Kingston University London, London, UK

**Keywords:** Pharmacogenomics, hospital pharmacists, pharmacogenomic testing, training, barriers

## Abstract

**Introduction:**

Pharmacogenomics (PG), the study of how genetic variations impact individual responses to drugs, has seen significant advancements globally in recent years. Hospital pharmacists play a crucial role in multi-disciplinary teams and understanding their preparedness to deliver PG services is essential for successful integration into the healthcare systems. This study evaluates their knowledge, training and seeks their views on PG testing implementation.

**Methods:**

A cross-sectional study was conducted on hospital pharmacists practising in Southwest London with the sample size determined as 137. The study was ethically approved. A structured, self-administered questionnaire was initially piloted, then distributed using emails with a link to Microsoft Form over a three-month period. It comprised 31 questions covering training levels, confidence, knowledge, perceptions, barriers to implementation and demographics.

**Results:**

A total of 46 responses were received achieving a response rate of 33.6%. The study revealed that 65% of participants had limited familiarity or understanding of PG. Over 50% indicated not receiving previous undergraduate or postgraduate training relevant to PG and accordingly their responses to the PG knowledge questions were lacking. Pharmacists with postgraduate training demonstrated better awareness and knowledge. An overwhelming number of participants envisaged carving a role for themselves favouring those that would complement their expertise in medicine management such as recommending appropriate treatment and dosages and suggestions based on PG testing results. Barriers identified were mostly concerning financial cost and shortage of trained staff to support PG services.

**Conclusions:**

Most surveyed pharmacists were not prepared to deliver PG services and thus require tailored training; nonetheless, they exhibited a positive attitude towards PG suggesting a willingness to bridge learning gaps. This presents an opportunity for relevant organisations to provide necessary training and for universities to enhance the curriculum enabling pharmacists to be involved in PG implementation.

## 1. Introduction

Pharmacogenomics (PG) investigates how an individual’s genetic makeup can affect their response to drugs. By identifying variations in genes that influence drug response, PG testing enables the prediction of therapeutic outcomes and reduces the occurrence of adverse events associated with genetic differences. The Human Genome Project (HGP) was one of the first initiatives in response to the ever-increasing demand to understand the genetic basis of human diseases where the first sequence of the human genome was generated (Collins & Fink, 1995). The significance of the HGP was the emergence of concrete understanding that laid the foundation of PG and the study of individuals’ responses to drugs based on genetic factors that influence the metabolism and distribution of drugs within the body (Benet et al., 2019).

Recognised for its impact of genetic variations on drug response, health authorities such as Health Canada and the Federal Drug Authority (FDA) have put PG biomarker information on the labels of more than 100 and 200 drugs, respectively. The recognition of variant genes’ ability to alter the metabolism of various drugs, and thereby their effect is an integral step in advancing PG testing and implementation (U.S. Food and Drug Administration, 2024). One of the leading studies that paved the way to PG was abacavir, an antiretroviral medication used in the treatment of human immunodeficiency viruses (HIV) (Mallal et al., 2002). It was found that individuals with an allele known as HLA-B*5701 are at the highest risk of developing hypersensitivity reaction. Consequently, recommendation was made to screen patients for HLA-B*5701 to minimise the risk of these adverse reactions, which can be fatal (Royal College of Physicians, 2022).

Another example is testing for gene variants in dihydropyrimidine dehydrogenase *(DYPD)* before starting cancer treatment with fluorouracil given by injection or infusion. This gene encodes for the enzyme dihydropyrimidine dehydrogenase (*DPD*) that catalyses the rate-limiting step in fluorouracil metabolism. *DPD* deficiency is most often caused by inherited variants of the *DYPD* gene and therefore patients with a known complete *DPD* deficiency must not be given fluorouracil injection or infusion as this puts them at higher risk of severe and life-threatening side effects (European Medicines Agency, 2020; Medicines & Healthcare products Regulatory Agency, 2020).

Clopidogrel, one of the most commonly prescribed antiplatelet drugs, is a prodrug that requires activation via the enzyme *CYP2C19*. Mutation of this enzyme, where a guanine at position 281 within the coding segment of the gene is replaced by an adenine, results in an allele is known as CYP2C19*2A that is not functioning (Brown & Pereira, 2018). Patients who carry one or two copies of the allele CYP2C19*2A are poor metabolisers and thus have reduced benefits from inhibition of platelet activation which occurs at a higher rate in certain ethnic groups (Lee et al., 2022). Accordingly, recent guidelines have suggested that clinicians should provide genotype testing to individuals prescribed clopidogrel to identify those with a genetic predisposition that affects their response to this drug, an approach deemed as being beneficial to patients and cost effective (National Institute for Health and Care Excellence (NICE), 2023).

Based on the growing evidence concerning the positive value of PG testing, the National Health Service (NHS) in the UK has set future targets for the implementation of PG across its services. The strategy has been outlined in a publication which focussed on embedding genomics and delivering equitable genomic testing for improved outcomes. The publication outlines the expectation of diagnostic testing centres to be introduced, which ultimately will require personnel who are trained and competent in handling laboratory samples, those with the ability to analyse and interpret the data generated, optimise treatments and provide counselling. This involves collaboration with diverse professional groups and academic institutions to provide a range of educational opportunities, spanning from raising awareness and informal learning to structured training and education (NHS England, 2022).

Pharmacists have always played an important role in personalised medicine, and genetic information offers additional means to improve this task. The role of pharmacists in genomics includes key activities such as ordering PG testing, identifying patients who may benefit from it, monitoring patients, providing drug information, and incorporating PG information into drug therapy (Royal Pharmaceutical Society, 2022). Hospital pharmacists in the UK are expected to be crucial in applying genomic medicine across several areas such as the use genomic data for antimicrobial stewardship allowing for precise antibiotic use, in cancer care and for managing rare genetic conditions like cystic fibrosis. Further roles for primary or secondary care pharmacists include discussing genetic testing results with patients, or ensuring CYP2C19 test results are obtained before patients begin treatment with clopidogrel, or identifying patients who may have a genetic condition, such as familial hypercholesterolaemia, for appropriate testing and follow-up management (Royal Pharmaceutical Society, 2023).

However, despite the potential benefits of PG testing in terms of patient safety and treatment efficacy, it is still not widely used in routine clinical care. The unfamiliarity of healthcare providers with PG, the cost of testing, time constraints, the absence of clear clinical guidelines, the lack of readily available tests, and various ethical considerations account for the slow uptake of PG in primary care (Klein et al., 2017; Rafi et al., 2020). Genomics’ specialists in the UK have identified several barriers to its implementation including technical challenges related to digital infrastructure for data interoperability and sharing. Additionally, involving end-users in the design and adoption of PG workflows is vital for the success of the process. Upskilling the workforce and promoting cultural change are essential for realising the benefits of PG-supported decision-making, including economic benefit for all stakeholders (The Academy of Medical Sciences, 2023).

From the review of current literature, it is evident that conducting this test in hospitals prior to commencing specific treatments benefits the patient. The rationale for our study is to determine the level of awareness and knowledge that hospital-based pharmacists possess regarding PG, their involvements in the PG services offered to date and their views on how they wish to embrace this service in the future.

## 2. Methods

### 2.1. Study design

A self-administered cross-sectional survey was used that consisted of 31 questions covering four key themes including: training pharmacists have received to date and their confidence regarding PG (11 questions), current knowledge/awareness and experience (9 questions), perceptions and view on PG future implementation (2 questions), barriers to implementation (2 questions) and finally demographics (7 questions). A variety of questioning styles were adopted to create a questionnaire which included open-ended and closed-ended questions, multiple choice questions and questions with 5-point Likert-type scales. The questions in this survey are similar to those utilised in a parallel study conducted by the authors concurrently, which sought the views of community pharmacists practising in the same geographical area on PG (Ghazal et al., 2023). Content validity was based on literature review to ensure that the questionnaire covered relevant aspects of PG identified in previous research and current expected knowledge in the field. The questionnaire was piloted prior to dissemination to the sample population and validity was derived from the pilot study feedback.

### 2.2. Sample size and participants

Pharmacists working in Southwest London hospitals were invited to participate in the survey using convenience sampling. According to the General Pharmaceutical Council register, there are 1009 pharmacists registered in that area. Sample size was calculated based on a 2019 survey which documented that approximately 21% of all pharmacists were hospital based, thus equating to 212 pharmacists. Raosoft’s calculator (Raosoft Inc., 2004) was employed for calculating sample size which yielded 137 (5% margin of error, 95% confidence level). The inclusion criteria were pharmacists practising in NHS hospitals located in the named region with no restriction on years of experience or speciality. The exclusion criteria were trainee pharmacists or pharmacists from other sectors such as academics or community.

### 2.3. Ethics

This study received an ethical approval in February 2023 from the delegated ethical approval team operating under the ethics committee of the Kingston University Faculty of Health, Science, Social Care and Education. All participant responses were anonymous and confidential; survey responses were kept in a password protected secure file, with only the researchers having access in line with General Data Protection Regulation (GDPR) requirements.

### 2.4. Data collection and analysis

Before commencing the full-scale data collection, a pilot study of the survey was sent to community pharmacists in the greater London area prior to carrying out the main study to assess its appropriateness. Subsequent feedback from the pilot study indicated that the survey was lengthy, prompting a reduction in the number of the questions and simplification of the survey. Accordingly, a simplified version of the survey was created. Hospital pharmacists from the specified area were invited to participate via emails which contained a link to the survey. This study also used snowball sampling to increase participation of eligible pharmacists. Additionally, to enhance response rate, some pharmacists were approached in person at their workplace and a QR code was provided to access the survey. All participants were provided with a participant information sheet which detailed the nature of the survey alongside considerations for contact, purpose of study and confidentiality. Data collection took place after obtaining ethics approval and continued until the end of April 2023.

Qualitative and ordinal data (such as Likert scale) were analysed using IBM SPSS software. Descriptive statistics, including percentages, frequencies, averages, and modes were used. Weighted averages were calculated for Likert-scale questions used within the study.

## 3. Results

### 3.1. Demographic information

A total of 46 hospital pharmacists completed the survey, giving a response rate of 33.6% (46/137). The gender distribution showed a distinct majority of females at 76.1% (35/46). Just over half of respondents were between the ages of 23–32, whilst only 1/46 was in the age range of 53–62. The number of respondents practising for up to 10 years was slightly higher at 59% (27/46) than those practising for more than 10 years at 41% (19/46). Both clinical and ward pharmacists were the main common roles held, at 32.6% (15/46) and 30.4% (14/46), respectively. Table 1 presents the demographic data of the respondents.

**Table 1.** Demographic details of respondents.

Characteristics	Number of participants (n = 46) n (%)
**Age Group**	
23–32	25 (54.3%)
33–42	13 (28.2%)
43–52	7 (15.2%)
53–62	1 (2.2%)
**Gender**	
Male	*11* (*23.9%)*
Female	35 (76.1%)
**Number of years in practice**	
< 1	2 (4.3%)
1–4	12 (26.1%)
5–10	13 (28.3%)
11–15	6 (13.0%)
16–20	6 (13.0%)
20+	7 (15.2%)
**Pharmacy role held**	
Clinical	15 (32.6%)
Dispensing	1 (2.1%)
Manager	7 (15.2%)
Specialist	5 (10.9%)
Training and education	4 (8.7%)
Ward	14 (30.4%)

### 3.2. Pharmacogenomic training and confidence level

Only 28.3% (13/46) of respondents indicated they were taught PG during their time at university, and only one of whom (1/13) rated this learning as ‘extremely useful’ for their current practice. One participant was motivated by self-interest to pursue specialised master’s degree in genomics. A similar number 26.1% (12/46) received postgraduate training about PG, of whom five (5/12) indicated the training was either useful or extremely useful. However, only one of those (1/5) stated that they strongly agree in their ability to confidently carry out PG testing, giving recommendations based on PG testing results. Three of the participants indicated receiving both education at university level and training afterwards.

The vast majority of participant expressed an overall low confidence in conducting PG testing. Only two participants (2/46) reported feeling confident in carrying out PG testing, providing health care professionals with recommendations based on PG testing results and explaining these results to patients.

**Figure 1.**
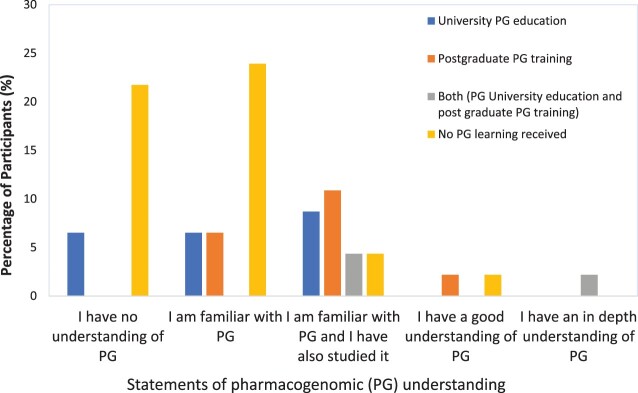
Pharmacists perceived understanding of PG topic in relation to their previous pharmacogenomics learning (n = 46).

In terms of familiarity with the concept of PG, the predominant response was indicative of a general lack of awareness and a limited of understanding of the topic. Figure 1 depicts the breakdown of the responses based on previous PG learning highlighting that individuals who reported not receiving any past PG learning tend to declare poor understanding or only a basic familiarity with the topic.

Regarding PG training, the most frequent mode of training received was webinar participation 58.3% (7/12) followed by e-learning 41.7% (5/12). Notably, of those who had received PG training, 42% (5/12) declared it was due to self-interest whereas 83.3% (10/12) stated their pursuance was driven by a combination of continuing professional education, self-interest and employer mandated.

Responses to an open-ended question about improving training varied among participants who had already received training. Four out of twelve advocated for re-building foundation knowledge due to perceived gaps from their university knowledge. Two participants recommended including a broad range of conditions as their training was limited to cancer or antibiotics treatments. Five participants requested more case studies and practical applications relevant to clinical practice. On the other hand, one participant highlighted the wealth of available resources and proposed that improvement can be achieved by better utilisation for these resources ‘I think that the available resources for NHS staff including pharmacists are excellent, readily and freely available and using an extremely wide range of teaching methods and media. The main way that it can be improved is by raising awareness of genomics and what is available’. Notably, only four out of all 46 respondents (8.7%) indicated they actually carried out PG testing services and these were for abacavir, fluorouracil, mercaptopurine and gentamicin.

In terms of future training preferences, participants were able to select more than one option. Nearly equal numbers of respondents chose online training 76.1% (35/46) and in-person training 73.9% (34/46). Shadowing an experienced PG pharmacist was preferred by 47.8% (22/46). Additionally, 52.2% (24/46) respondents expressed a preference for in-depth university teaching, while 13/46 preferred specialised master courses. Additionally, it has been suggested to include ‘workplace-based competency training’ and having readily available and accessible resources for addressing clinical practice needs arising from patients’ test results.

### 3.3. Knowledge, awareness and experience

Only one of the participants (1/46) indicated that they have an in-depth understanding of PG and expressed confidence in carrying out PG testing, giving health care professionals recommendations based on PG testing results, explaining PG testing results to patients and accurately applying the results of PG testing to drug therapy selection, dosing and monitoring.

Notably, of the 4.4% (2/46) of respondents who indicated a good understanding of PG, one stated their neutrality on their ability to carry out all PG testing relevant activities, while the other participant believed they would be confident in all these activities.

More specific knowledge was sought by asking if respondents could choose the correct enzyme responsible for metabolising warfarin. Certain genetic variants of the enzyme ‘CYP2C9’ result in a reduced ability to metabolise this drug leading to over-anticoagulation and bleeding. Overall, 45.7% (21/46) correctly identified the enzyme responsible. The group that scored the best at 33.3% (7/21) was those qualifying between 5 and 10 years. Of the 13 respondents who received PG relevant education at undergraduate level, 53.8% (7/13) correctly identified the enzyme. Those with who received postgraduate training did much better at 75% (9/12), more than doubling those who did not pursue training at 35.3% (12/34). The Chi-square was applied to evaluate if the there is an association between training and ability to answer this question correctly, it showed a *p*-value of 0.0176 and therefore the results are significant at *p* < .05.

Respondents’ knowledge regarding PG was further explored by asking them to categorise common drugs encountered in practice into those earmarked for future PG testing, or currently having PG testing available, or neither. The rationale was to gauge their general knowledge about PG topic, which is expected to be acquired through university education or professional training and continuing professional development (CPD) among respondents who received postgraduate training on PG, 66.7% (8/12) and 50% (6/12) correctly identified the PG status for fluorouracil and abacavir, respectively.

Concerning the years of experience, those with less than 10 years of experience demonstrated slightly better knowledge when asked about fluorouracil current PG status at 63.6% (7/11) compared to longer practising pharmacists. All participants who received both undergraduate and postgraduate training identified fluorouracil status (3/3), followed by the group that received postgraduate training only (5/9). Conversely, only 2/22 participants who indicated not receiving any previous education or training answered correctly. The Fisher’s Exact test (2 × 2) revealed a significant relationship between PG education and training and the ability to show specific knowledge represented by answering this question correctly, with a value of 0.0149 (*p* < .05). Furthermore, it is important to note that those who did not receive undergraduate PG education or postgraduate training exhibited poor PG knowledge across all the drugs.

### 3.4. Perceptions and views for future implementation

When participants were asked to select various roles within PG testing services that they envisage to offer, 89.1% (41/46) expressed a preference for making medicine recommendations based on results as well as recommending doses and monitoring. This was followed by 73.9% (34/46) who favoured interpreting test results, and 58.7% (27/46) stated the role of requesting PG testing. Meanwhile, conducting laboratory analysis or taking samples were the least favourable options with only 8.7% (4/46) participants choosing these roles.

Respondents viewed the role of PG in improving patient outcomes and increasing safety in terms of adverse drug reactions (84.8%, 39/46 for each). Table 2 summarises their views on the benefits of PG implementation.

**Table 2.** Respondents’ views on benefits of PG implementation.

Benefits	% Responses (n = 46)
Cost control of drug therapy	60.9% (28/46)
Improvement of patient care and satisfaction	69.6% (32/46)
Improved patient outcomes	84.8% (39/46)
Increased safety in terms of adverse effects reduction	84.8% (39/46)
Prevention of drug-drug interactions	63.0% (29/46)
Reduce hospital admission/re admission	65.2% (30/46)

In one of the open-ended responses where pharmacists could express their view about PG, one respondent showed a positive outlook for PG future ‘I do believe PG will be mainstream one day in every part of drug use and point of care testing will be an enormous development’. Meanwhile another respondent was sceptical about the usefulness of PG testing in clinical practice for drugs like warfarin and clopidogrel in terms of dosing or prescribing. Instead, the respondent recommended focusing on the drugs where benefits can be seen with pharmacogenomic testing. This comment may be influenced by findings from some economic evaluation studies indicating that PG testing for these drugs has not proven to be cost effectiveness (Verbelen et al., 2017).

### 3.5. Barriers to PG implementation

**Figure 2.**
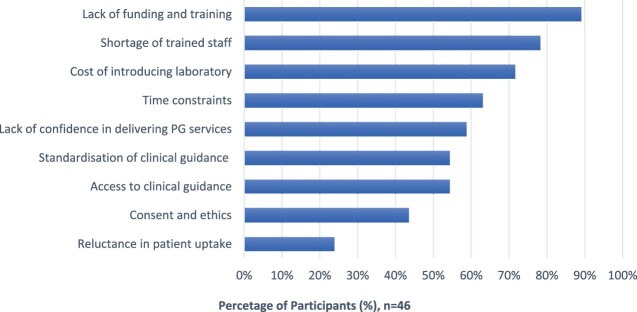
Pharmacists’ views on barriers to implementing PG testing service (n = 46).

When pharmacists were asked about the barriers they foresee potentially hindering or delaying the implementation of PG, 89.1% (41/46) highlighted a lack of funding and training as the predominant concern. Furthermore, 78.3% (36/46) of respondents viewed a shortage in adequately trained staff as another obstacle to service implementation. Figure 2 provides a summary of the perceived barriers reported by respondents.

## 4. Discussion

Although the study recruited a relatively small sample size, which may limit the ability to generalise the findings to the broader population of hospital pharmacists, the data still offers valuable snapshot of the current situation within the specific area of Southwest London hospitals, reflecting the views and practices of those who participated. This study highlights that despite having a positive outlook on PG and its associated importance towards improvement of drug therapy and reduction of side effects, few respondents have been involved in the PG process itself. This correlates with the findings of a European study conducted in 2017, where 34.3% of respondents did not order or recommended a pharmacogenomic test in the year prior to the survey (Just et al., 2017). The picture was slightly better in a study from the United States, which showed that approximately 50% of physicians surveyed ordered genetic testing in their clinics attributing this low uptake to limited or no training in genetics (Schaibley et al., 2022).

Participants in our study demonstrated their willingness to embrace PG via their validation of the various PG service provision roles they wish to take part on in the future. Despite their willingness, the slow integration of PG into clinical practice seems to be hindered by the lack of formal or mandated education surrounding PG in the pharmacy curriculum and postgraduate education, as identified a previous study where 40% of HCPs admitted they lacked PG expertise (Just et al., 2017).

In our study, 28.3% of respondents benefitted from PG undergraduate education but they did not report perceived level of understanding or knowledge, raising a question into the effectiveness or the retention of this teaching. This could possibly be due to the pharmacy curriculum not being standardised. Indeed, a review of the curricula of 22 pharmacy degree programmes in Australia revealed the scope of training was limited, and schools of pharmacy were covering basic concepts, such that teaching was at an ‘understand’ level (Venugopal et al., 2022). Pharmacy students highlighted the necessity for a revised pharmacy curriculum that prioritises PG and incorporates the testing into their curriculum (Arafah et al., 2022; Surofchy et al., 2017).

The General Pharmaceutical Council, regulating the pharmacy degree in the UK, has now mandated PG to be a learning outcome ‘Apply the principles of clinical therapeutics, pharmacology and genomics to make effective use of medicines for people, including in their prescribing practice’ where undergraduates are typically expected to progress from demonstrating ‘shows how’ to reaching ‘does’ level by the foundation training year, as defined by the Miller’s triangle which outlines the hierarchy of clinical competency (General Pharmaceutical Council, 2021; Miller, 1990). Accordingly, in line with the recognition that PG should be taught as an integral part of a healthcare curricula, this ultimately should help close the PG knowledge gap (Karas Kuželički et al., 2019).

Our study revealed a moderately low level of knowledge in PG with only 45.7% managed to correctly identify the enzyme responsible for the metabolism of warfarin, a very well-known drug in the world of pharmacy. Moreover, one fifth of the respondents on average were able to accurately identify drugs currently requiring PG testing. However, this figure is higher for those who received postgraduate training with an average score of 50%. A similar study (Jarrar et al., 2021), involving 370 pharmacists practising across various sectors found that 38% of respondents correctly listed medications requiring PG testing. Overall, pharmacists with postgraduate training performed the best when identifying drugs that have the potential for PG testing. However, this was still disappointing, considering there was a Drug Safety Update in October 2020 by the MHRA highlighting the need to test for *DPYD* gene before initiating treatment with injectable fluorouracil to identify patients at increased risk of severe and fatal toxicity (Medicines & Healthcare products Regulatory Agency, 2020).

In the present study, only four respondents out of 46 have carried out PG testing, yet an overwhelming number envisaged carving a role for themselves in the future. Given that the participants were clinical hospital pharmacists, they favoured roles that would complement their expertise in medicine management (89%) such as recommending appropriate treatment and dosages and suggestions based on PG testing results. This finding contrasts with our previous study on community pharmacists recruited from the same area who viewed their role as primarily involving taking samples (84%) and followed by medicine recommendations (60%) (Ghazal et al., 2023). This shows that practitioners tend to gravitate towards roles they are familiar with and that are integrated into their daily practice.

There was a widespread appreciation of the benefits of PG across the board from participants, including those who had not received training previously. In particular, 84.8% of respondents saw PG as beneficial to improve patient outcomes and increase safety by reducing adverse drug reaction. This suggests that most respondents perceive PG as a positive tool with the capacity to benefit patients regardless of their level of knowledge or awareness.

The low participation in PG delivery to date, at 8.7% did not deter respondents to voice their opinions on a number of barriers to implementation. Top of the list was lack of funding and shortage in trained staff. Respondents in this study demonstrated a realistic perspective by acknowledging the limited PG testing in routine clinical care in the UK. The unfamiliarity of healthcare providers with PG, the lack of resources including clear clinical guidelines, necessary funds, readily available tests, time constraints, and various ethical concerns were listed as barriers to PG implementation which aligns with findings of previous (Kabbani et al., 2023). Just over half of the respondents perceived resources as a barrier. Having access to clinical guidance or decision-making pathways will help standardise result interpretation and making PG implementation a smoother process, especially when there is currently a lack of understanding on how to use the results of genetic screening to aspire to precision medicine prescribing (Caudle et al., 2016).

Reluctance in patient uptake was perceived as the lowest barrier at 23.9%. Over the past decade, patients have become more involved in their own healthcare decisions. A study revealed that nearly half of the participants had poor knowledge and low awareness of PG within their care. However, when the pharmacogenetic concept was explained, there was a two-fold increase in acceptance (O’Shea et al., 2022).

To prepare pharmacists to effectively fulfil this emerging role for the provision of PG service, it is essential to incorporate dedicated PG learning into the pharmacy curriculum focused on explaining the science behind PG as well as its clinical applications. Nevertheless, the survey findings showed that pharmacists who received PG postgraduate training had a better knowledge in PG including those who received PG education at the university level. A combination of both, undergraduate and postgraduate education potentially can have a synergistic outcome. This underpins the importance of developing continuous professional development courses in PG. This initiative has already been taken by the Health Education England, Genomics Education Programme, as well as the RPS and the Centre for Pharmacy Postgraduate Education in the UK, as PG increasingly integrate into mainstream of the NHS care. Moreover, as emphasised by one of the respondents, it is more crucial to promote these learning or even mandate them to ensure pharmacists ready to contribute to the delivery of the service.

In summary, the findings of our study contribute to understanding the current landscape of pharmacogenomics implementation in hospital settings in the UK and highlight the inadequate readiness of the surveyed hospital pharmacists. Key findings included the current low uptake of PG testing, the need of mandated and engaging training and targeted educational initiatives. Additionally, the study revealed perceived barriers but also identified facilitators, particularly the willingness of hospital pharmacists to be involved in genomics integration. Considering the NHS plans to accelerate the use of genomic medicine, our findings suggest that their policy should prioritise equipping the workforce to be ready to deliver this service.

### 4.1. Limitations and improvements

The study encountered some limitations. Firstly, there was a low response rate leading to a small sample size and therefore, the results may be underpowered and cannot be generalised to a larger population. This is possibly due to the typically busy nature of hospital pharmacists. Additionally, the survey included complex questions, particularly in the knowledge section, which might have been challenging for some pharmacists to answer. Moreover, considering potential regional differences in the implementation of pharmacogenomics (PG), it would have been beneficial to gather input from hospital pharmacists nationwide who have a broader perspective of direct experience with PG testing services.

## 5. Conclusions

The emergence of PG as a clinical tool to optimise drug therapy has captured the attention of healthcare practitioners. Yet PG implementation has been a slow road, even within the hospital setting. Pharmacists in this study displayed a positive outlook to embracing this service however their specific knowledge was moderately low. The knowledge was improved in those who upskilled themselves post-graduation, posing the question of whether PG training should be made mandatory. With the General Pharmaceutical Council mandating the inclusion of genomics teaching in pharmacy courses in the UK, it would be of interest to revisit this in the coming years to explore the impact of enriched curricula on pharmacists’ knowledge and confidence.
